# Ear mold for congenital ear malformation: A randomized controlled trial

**DOI:** 10.1097/MD.0000000000021313

**Published:** 2020-07-24

**Authors:** Kun Zou, Yanjun Fan, Lihua Jiang, Jincheng Huang, Yunqi Miao, Chunsong Yang, Min Yang, Li Zhao

**Affiliations:** a Department of Health Policy and Management; b West China Research Centre for Rural Health Development, West China School of Public Health and West China Fourth Hospital, Sichuan University, Chengdu, Sichuan; c National Center for Women and Children's Health, China Center for Disease Control and Prevention, Beijing; d Department of Health-Related Social and Behavioral Sciences; e Department of Epidemiology and Biostatistics, West China School of Public Health and West China Fourth Hospital, Sichuan University; f Department of Pharmacy, West China Second Hospital, Sichuan University; g Evidence-Based Pharmacy Center, West China Second Hospital, Sichuan University, Chengdu, Sichuan, China; h Faculty of Health, Art and Design, Swinbune Technology University, Melbourne, Australia.

**Keywords:** congenital auricular deformities, ear mold, randomized controlled trial

## Abstract

**Background:**

Congenital auricular deformities (CAD) are prevalent worldwide. The objective of this study is to investigate the effectiveness and safety of ear molding for children with CAD at their early days.

**Methods:**

One hundred and nighty children (under 3 days) with CAD will be included in the study. Participants will be randomly allocated to treatment or waiting list group (n = 95). The treatment group will receive ear molding within 3 days after birth for 2 weeks. The control group will receive usual care and receive the same ear molding at 6th week if spontaneously recover is not occur. Physician and parent assessment of improvement, parent's anxiety, depression, and quality of life and adverse events will be measured at baseline, 3rd and 6th week of initial treatment. The primary outcome recovery rate will be compared between groups using Chi square test. Secondary continuous outcomes will be compared using analysis of variance.

**Discussion:**

This study is the first randomized controlled trial to examine the effectiveness, safety and cost-effectiveness of ear molding for CAD comparing with waiting list, to inform clinical decision of CAD treatments and relevant guideline development.

## Abstract 2

Supplemental Digital Content is available in the text

## 1 Introduction

Congenital auricular deformities (CAD) or congenital ear malformation are common in children. However, the prevalence of CAD is varied surprisingly from 1.7% to 50% among different populations,^[1–5]^ which may at least partially due to definition and diagnosis criteria. CAD will not only affect the appearance, but also hearing and mental health of the child. Parents’ mental health and quality of life may also be affected. Observational study has shown that about 70% infants with CAD at birth will recover naturally at 1 years old, but who will recover spontaneously is unclear.^[3]^

Traditional treatments for CAD are mainly surgical, which normally be arranged at around 6 years old.^[6]^ They are invasive and painful by its nature, and complications are not rare,^[7]^ including hematoma, infection, bleeding, allergic reactions, and necrosis, hypertrophic scars, keloids, fistulae, and recurrence.^[8]^ What is more, impairment of hearing or mental health of the child affected may already happened, which may have life-long consequences.^[9,10]^ Additionally, surgeries and subsequent care are expensive, bringing considerable economic burden to the family and society.

Non-surgical interventions of CAD were initialized in the 1980s.^[11]^ They are attractive for many health professionals and patients for the noninvasive nature, relatively easy process, sound curative rate and low cost compared with surgeries. At the primitive stage, doctors used tape and stick to shape the ear of children with CAD and found promising results, but highly relied on the skills of the doctors.^[12,13]^ Tape and stick were evolved to ear molding which increased the standardization of the treatment in recent decade.^[14]^ Studies found that the effectiveness rate of ear molding for CAD was up to 90%, and earlier intervention could lead to better outcomes, with few adverse events.^[15–19]^ However, these studies are mainly observational study or noncontrolled study, fall short of methodological rigour and prone to biases. As far as we know, there has been no randomized controlled trial (RCT) evaluating the effectiveness, safety and cost-effectiveness of ear molding for CAD and.

## 2 Objective

The objective of this study is to evaluate the effectiveness, safety and cost-effectiveness of ear molding for CAD.

## 3 Methods

### 3.1 Study design and setting

A multicenter, assessor-blind, parallel 2 arms RCT will compare ear molding with waiting list in children with CAD who are in attendance in 8 woman and child hospitals in cities including Chengdu, Chongqing, Zhengzhou in China. The study has been submitted to the Ethnic Committee of Medical Research of West China Medical Centre, Sichuan University (K2020016) and was registered in the Chinese Clinical Trial Registry (ChiCTR2000031052) 21 March 2020.

The protocol is in accordance with the 2013 SPIRIT (standard protocol items: Recommendations for Interventional Trials) statement.^[20,21]^ The procedure of the trial is presented in Table 1.

**Table 1:**
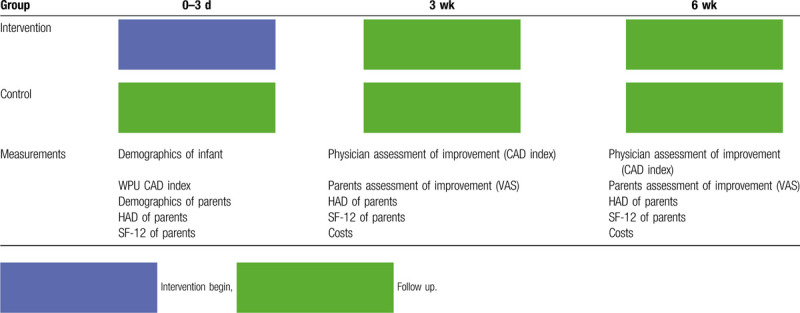
Process of the trial.

### 3.2 Participants

#### 3.2.1 Inclusion criteria

The inclusion criteria recommended by the expert consensus on the ear molding for congenital auricular deformation made by the Subspecialty Group of Pediatrics, Society of Otorhinolaryngology Head and Neck Surgery, Chinese Medical Association was adopted.^[22]^ The inclusion criteria are:

(1) Children aged 0 to 3 days,
(2) Diagnosis of auricle morphological abnormalities, including: prominent ear, Stahl's ear, top ear, cup ear, cryptotia, abnormal convex ear nails Conchal crus, helical rim deformity, mixed ear deformities combining 2 or more deformities, and other auricle distortions;
(3) Diagnosis of Microtia Marx classification I degree.^[23]^

#### 3.2.2 Exclusion criteria

(1) Low birth weight infants (weighing less than 2500 g),
(2) with multiple organ deformities,
(3) Acute dermatitis,
(4) Patients with other health-related reasons that are not suitable for participation in the study.

### 3.3 Recruitment

Children born at the Department of Obstetrics of participant hospitals will be examined by health professionals against CAD within 3 days in the department. Parents of children diagnosed with CAD and eligible for the inclusion and exclusion criteria will be invited to participate into the trial. Children whose parents signed the informed consent form will be included in the study.

### 3.4 Randomization and allocation concealment

A biostatistician will generate the random sequence using computer program. Central randomization will be performed by the biostatistician to achieve allocation concealment. Each study center will recruit participants according to the inclusion and exclusion criteria and obtain the informed consent from participants’ parents. After completing the demographic data collection, baseline assessment and baseline data entry, study center will contact the biostatisticians to obtain a grouping code to randomly assign the participant to either the intervention or the control group.

### 3.5 Intervention

Participants in the intervention group will receive ear molding delivered by health professionals within 3 days of birth besides usual neonatal care, with a treatment duration of 2 weeks and followed up until 6 weeks from the start of the treatment.

### 3.6 Control

The control group will be waiting list and receive usual neonatal care. Participants will be followed up at the same intervals as the intervention group until the sixth week after birth. After that, participants will be offered ear molding treatment as the same as the intervention group according to parents’ will, if the participant is not recovered spontaneously.

### 3.7 Treatment monitoring and discontinuation

Since the starting of ear molding, participants will be seen once a week by the responding health professional to check and assure the mold is in the right place. Any adverse events will be treated and medical advices will be given accordingly, including suspension or discontinuation of ear molding. Other treatments of CAD received by the participants will be asked and recorded in the case record form (CRF) at each point of follow up. Vouchers will be offered to participants who complete follow up.

Participants can withdraw from the trial on any reason, which will not affect their treatments or usual care. In addition, patients who withdraw due to adverse events will be treated and monitored until recovery.

### 3.8 Outcome measures

#### 3.8.1 Primary outcome

*Physician assessed improvement.* Two doctors who do are unaware of the group assignment of participants will assess the improvement of auricular form independently. Standardized photographs of participants’ ears will be obtained and assessed using the West China-Peking Union (WPU) index for CAD designed by the research team for the study (see supplementary Table 1 and supplementary Figure 1). Standardized photographs of ears will be obtained by the following way:

(1) Photographs will be obtained at the following anatomical position: upright (showing binaural), fully lateral (showing monaural), lateral 45 degrees (showing monoaural), posterior (showing binaural), and axial position (showing binaural) Among them, the full lateral position and the lateral 45-degree position are about 25 cm from the ear surface;
(2) both ears will be photographed in the lateral position and the lateral 45-degree position to facilitate the comparison of the auricle shape;
(3) Photographs should clearly and fully show the shape of the auricle, multiple photographs at one angle (1–3 photographs) can be taken for this purpose.

The average score of the 2 physicians’ assessment will be used to calculate the improvement value using the following formula: Improvement value = (total follow-up score — total baseline score) / total baseline score.

The effect size of the improvement value of 0.1 or less will be defined as ineffective, 0.1 to 0.3 as small, 0.3 to 0.5 as medium, and >0.5 as significant. Ineffective and small effect will be classified as nonsignificant improvement (0), and medium or significant effect will be classified as significant improvement (1), turning the effect size into a dichotomous variable, upon which the improvement rate will be calculated.

#### 3.8.2 Secondary outcomes

*WPU CAD index score.* Two physicians will evaluate ear abnormality of participants using the CAD index to obtain a total score, ranges from 0 to 100 from the worst to the best. The final score will be the average of the two physicians’ assessment score.

*Parents’ evaluation of improvement.* Parents of participants will use a visual analogue scale to evaluate the ear abnormality of participants, where 0 represents no improvement and 10 represents complete recovery to normal.

*Anxiety and depression of parents.* The hospital anxiety and depression scale (HADS) will used to evaluate the parents’ anxiety and depression.^[24,25]^

*Quality of life of parents.* The self-administered 12-Item Short Form will be used to assess the quality of life of parents.^[26]^

*Adverse events.* Any adverse events will be recorded in the CRF including but not limited to: skin swelling, skin lesions, allergies, eczema, local infections, necrosis, recurrence, or other unforeseen mild or severe adverse events.

*Time of measurement.* The primary and secondary outcomes, together with any adverse event will be measured and recorded in the CRF at 3 and 12 weeks after the treatment.

*Cost.* The costs will be collected from society perspective, which includes both direct and indirect costs.^[27]^ Direct medical costs will include:

(1) outpatient and inpatient costs: orthosis costs, treatment costs, and examination costs. Since the wearing time limit of each brace is 2 weeks, and there is a possibility of rewearing due to relapse, the number of braces used during one ear treatment can exceed 2, thus the number of braces used will be recorded.
(2) The cost of managing complications and adverse reactions.

Indirect medical costs will include: costs incurred by inviting the person to take care of the patient, time lost for family members to take care of the patient, as well as transportation to and from the hospital. Cost-effectiveness analysis will be conducted for the primary outcome physician assessed improvement.

### 3.9 Blinding

Due to the nature of ear molding, it is impossible to blind the interventionist, participants and their parents. Therefore, assessor blinding will be used for the primary outcome physician assessment of improvement and secondary outcome WPU index score.

### 3.10 Sample size

The test level α is 0.05, and the statistical power is (1- β) is 0.9. According to literature reports, the effective rate of ear mold correction within 2 weeks of age was about 90%, and the natural improvement rate without intervention was about 70%. The sample content was calculated to be 79 cases per group. According to the possible loss of follow-up of 20%, the sample was expanded to 95 cases in each group, with a total of 190 cases in both groups.

### 3.11 Data management

The data will be recorded in the CRFs and input into an online database within 2 days of the follow up, which include participant demographics, disease histories, physician assessment of improvement, parents’ self-administrative questionnaires of HADS and 12-item short form survey of quality of life of quality of life. CRFs will be input into the database by 2 independent persons and cross-checked. The database will be locked when data input is finished and checked for data analysis by a biostatistician not aware of the group allocation of patients. The participants data will be protected and only used for this study. Personal information of participants will be kept confidential and anonymous data will be used for analysis. The principle investigator has access to the final trial dataset.

### 3.12 Statistical analysis

For descriptive analysis, mean and standard deviation will be used for symmetric continuous variables, while median, first quartile, and third quartile will be used for asymmetric continuous variables. Frequency and percentage will be used to describe categorical variables. In baseline analysis of comparability between groups, analysis of variance will be used for continuous variables and Chi square test will be used for categorical variables.

The primary outcome improvement rate will be compared between groups and risk ratio with 95% confidence interval will be estimated using multi-level modelling adjusting for individual and hospital level factors. For secondary outcomes, mean difference with 95% confidence interval of WP CAD index score, parents’ evaluation of improvement (visual analogue scale), anxiety and depression, quality of life using multi-level modelling. Potential confounding factors at individual and study site level, and secular trend will be included/adjusted in the models. Intent to treat analysis will be conducted for the effectiveness analysis. Sensitivity analysis will be carried out using per protocol analysis. The significant level will be at 0.05. Statistical analysis will be performed using Stata 15.0 SE and MLN.

## 4 Discussion

This study is the first RCT to examine the effectiveness and safety of ear molding for CAD comparing with waiting list, and will provide cost-effectiveness evaluation of this treatment strategy to inform clinical decision of CAD treatments and relevant guideline development.

## Acknowledgments

The authors would like to thank our colleagues and staff at Chongqing Health Center for Women and Children, Sichuan Province Hospital for Women and Children, Chengdu Women and Children Hospital, Chengdu Angel Women and Children Hospital and Henan Province Hospital for Women and Children for their support. We also would like to thank Professor Leren He from the Plastic Surgery Hospital, Chinese Academy of Medical Sciences, Peking Union Medical College for suggestions and comments on the study design and measurement tool and Dr. Qinhao Gu from the Plastic Surgery Hospital, Chinese Academy of Medical Sciences, Peking Union Medical College for providing the photograph of anatomical structures of the ear.

## Author contributions

Conceived the study: LZ, YJF; study design: LZ, MY, YJF, JYL, KZ, LRH, CSY; will perform the study: LZ, YJF, KZ, MJH, YQM; will collect and manage the data: LZ, KZ, MJH, YQM; will perform the statistical analysis: KZ, MJH, YQM; wrote the draft: KZ, LZ, YJF, MY. All authors contribute to the revision and approved the final manuscript.

## Supplementary Material

Supplemental Digital Content

## Supplementary Material

Supplemental Digital Content
