# Supplementary figures and images for: Ear mold for congenital ear malformation: A randomized controlled trial
Source: Medicine (Baltimore). 2020 Jul 24;99(30):e21313. doi: 10.1097/MD.0000000000021313 (PMC7387052; doi:10.1097/MD.0000000000021313)

Supplementary Figure 1

**
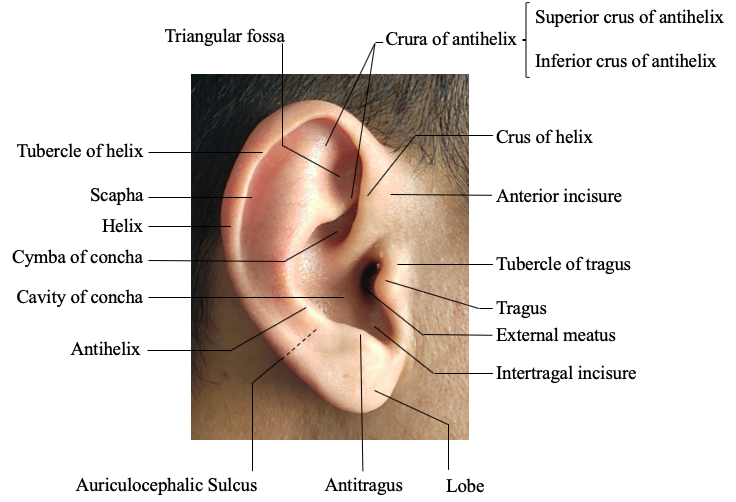
**

# Figure 1 Anatomical structures of the ear

Supplement: Supplemental Digital Content [file medi-99-e21313-s001.docx]
