# Supplementary material for: Ear mold for congenital ear malformation: A randomized controlled trial
Source: Medicine (Baltimore). 2020 Jul 24;99(30):e21313. doi: 10.1097/MD.0000000000021313 (PMC7387052; doi:10.1097/MD.0000000000021313)
Supplement: Supplemental Digital Content [file medi-99-e21313-s002.docx]

Supplementary Table 1

# Table 1 West China & Peking Union (WPU) index for congenital auricular deformities

| Anatomical structure of ear | Abnormity | Type | Abnormity degree |
| --- | --- | --- | --- |
| 1. Crura of antihelix | No Yes | Missing, Defects, Deformation | Minor, Mild, Medium, Severe, Very Severe |
| 2. Triangular fossa | No Yes | Missing, Defects, Deformation | Minor, Mild, Medium, Severe, Very Severe |
| 3. Crus of helix | No Yes | Missing, Defects, Deformation | Minor, Mild, Medium, Severe, Very Severe |
| 4. Anterior incisure | No Yes | Missing, Defects, Deformation | Minor, Mild, Medium, Severe, Very Severe |
| 5. Tubercle of tragus | No Yes | Missing, Defects, Deformation | Minor, Mild, Medium, Severe, Very Severe |
| 6. Tragus | No Yes | Missing, Defects, Deformation | Minor, Mild, Medium, Severe, Very Severe |
| 7. External meatus | No Yes | Missing, Defects, Deformation | Minor, Mild, Medium, Severe, Very Severe |
| 8. Intertragal incisure | No Yes | Missing, Defects, Deformation | Minor, Mild, Medium, Severe, Very Severe |
| 9. Lobe | No Yes | Missing, Defects, Deformation | Minor, Mild, Medium, Severe, Very Severe |
| 10. Artitragus | No Yes | Missing, Defects, Deformation | Minor, Mild, Medium, Severe, Very Severe |
| 11. Posterior sulcus | No Yes | Missing, Defects, Deformation | Minor, Mild, Medium, Severe, Very Severe |
| 12. Antihelix | No Yes | Missing, Defects, Deformation | Minor, Mild, Medium, Severe, Very Severe |
| 13. Concha | No Yes | Missing, Defects, Deformation | Minor, Mild, Medium, Severe, Very Severe |
| 14. Helix | No Yes | Missing, Defects, Deformation | Minor, Mild, Medium, Severe, Very Severe |
| 15. Scapha | No Yes | Missing, Defects, Deformation | Minor, Mild, Medium, Severe, Very Severe |
| 16. Tubercle of helix | No Yes | Missing, Defects, Deformation | Minor, Mild, Medium, Severe, Very Severe |
| 17. Vertical distance of cephaloauricular | | ________ mm | Minor, Mild, Medium, Severe, Very Severe |
| 18. Cephalo otic angle | | ________degree | Minor, Mild, Medium, Severe, Very Severe |
| 19. Overall assessment of deformities | Prominent ear, Stahl's ear, top ear, cup ear, cryptotia, abnormal convex ear nails Conchal crus, helical rim deformity, mixed ear deformities, others____ | | Minor, Mild, Medium, Severe, Very Severe |
| Total score*： Signature： DD/MM/YYYY: | | | |

Note：No, Minor, Mild, Medium, Severe and Very Severe scores 0, 1, 2, 3, 4, 5，respectively;

Total score = Σ（Item1~18）+ Item19*2 (Range 0~100)
